# Sil1-deficient fibroblasts generate an aberrant extracellular matrix leading to tendon disorganisation in Marinesco-Sjögren syndrome

**DOI:** 10.1186/s12967-024-05582-0

**Published:** 2024-08-23

**Authors:** Laura Amodei, Anna Giulia Ruggieri, Francesca Potenza, Marianna Viele, Beatrice Dufrusine, Raffaella Franciotti, Laura Pietrangelo, Matteo Ardini, Liborio Stuppia, Luca Federici, Vincenzo De Laurenzi, Michele Sallese

**Affiliations:** 1 Department of Innovative Technologies in Medicine and Dentistry, Chieti, Italy; 2 Center for Advanced Studies and Technology (CAST), Chieti, Italy; 3 Department of Bioscience and Technology for Food Agriculture and Environment, University of Teramo, Teramo, 64100 Italy; 4 Department of Neuroscience, Imaging and Clinical Science, Chieti, Italy; 5 Department of Medicine and Aging Sciences, Chieti, Italy; 6 Department of Life, Health and Environmental Sciences, University of L’Aquila, L’Aquila, 67100 Italy; 7 Department of Psychological Health and Territorial Sciences, “G. d’Annunzio” University of Chieti-Pescara, Chieti, 66100 Italy

**Keywords:** Endoplasmic reticulum, ER stress, Extracellular matrix, Unfolded protein response, Collagen, Cytoskeleton, Transcriptomic analysis, DQ-collagen, Electron micrograph, Wound-healing, Cell motility, Cell attachment, Woozy mouse, Skeletal muscle, Tendon

## Abstract

**Background:**

Marinesco-Sjögren syndrome (MSS) is an autosomal recessive neuromuscular disorder that arises in early childhood and is characterized by congenital cataracts, myopathy associated with muscle weakness, and degeneration of Purkinje neurons leading to ataxia. About 60% of MSS patients have loss-of-function mutations in the SIL1 gene. Sil1 is an endoplasmic reticulum (ER) protein required for the release of ADP from the master chaperone Bip, which in turn will release the folded proteins. The expression of non-functional Sil1 leads to the accumulation of unfolded proteins in the ER and this triggers the unfolded protein response (UPR). A dysfunctional UPR could be a key element in the pathogenesis of MSS, although our knowledge of the molecular pathology of MSS is still incomplete.

**Methods:**

RNA-Seq transcriptomics was analysed using the String database and the Ingenuity Pathway Analysis platform. Fluorescence confocal microscopy was used to study the remodelling of the extracellular matrix (ECM). Transmission electron microscopy (TEM) was used to reveal the morphology of the ECM in vitro and in mouse tendon.

**Results:**

Our transcriptomic analysis, performed on patient-derived fibroblasts, revealed 664 differentially expressed (DE) transcripts. Enrichment analysis of DE genes confirmed that the patient fibroblasts have a membrane trafficking issue. Furthermore, this analysis indicated that the extracellular space/ECM and the cell adhesion machinery, which together account for around 300 transcripts, could be affected in MSS. Functional assays showed that patient fibroblasts have a reduced capacity of ECM remodelling, reduced motility, and slower spreading during adhesion to Petri dishes. TEM micrographs of negative-stained ECM samples from these fibroblasts show differences of filaments in terms of morphology and size. Finally, structural analysis of the myotendinous junction of the soleus muscle and surrounding regions of the Achilles tendon revealed a disorganization of collagen fibres in the mouse model of MSS (woozy).

**Conclusions:**

ECM alterations can affect the proper functioning of several organs, including those damaged in MSS such as the central nervous system, skeletal muscle, bone and lens. On this basis, we propose that aberrant ECM is a key pathological feature of MSS and may help explain most of its clinical manifestations.

**Supplementary Information:**

The online version contains supplementary material available at 10.1186/s12967-024-05582-0.

## Background

Marinesco-Sjögren’s syndrome (MSS) is a rare autosomal recessive genetic disorder that occurs in early childhood [1]. The hallmark of MSS is congenital cataracts, muscle weakness and ataxia resulting from myopathy and Purkinje cell degeneration [2, 3]. Other distinguishing characteristics include mild to severe mental impairment, hypergonadotrophic hypogonadism, short stature and skeletal abnormalities [2]. Approximately 60% of MSS patients have homozygous or compound heterozygous mutations in the SIL1 gene [4]. This gene encodes an ATP exchange factor for the immunoglobulin binding protein (BiP) [5]. The folding of newly synthesised proteins involves the ATPase cycle of BiP, therefore the loss of Sil1 impairs both the BiP cycle and protein folding [2, 6]. Specifically, BiP remains associated with its client proteins, causing the accumulation of unfolded proteins, endoplasmic reticulum (ER) stress and activation of the unfolded protein response (UPR) [2, 6].

The UPR activates a signalling and transcriptional programme aimed at restoring ER homeostasis. This pathway reduces cellular RNA and protein synthesis, increases ER folding capacity and improves the elimination of unfolded proteins through ER-associated degradation (ERAD) [7]. Three ER transmembrane proteins participate into the UPR: the PRK-like endoplasmic reticulum kinase (PERK), the inositol-requiring enzyme 1 (IRE1), and the activating transcription factor 6 (ATF6) [8]. PERK, by phosphorylating eukaryotic translation initiation factor 2 A (eIF2α), attenuates protein synthesis and promotes the expression of several proteins, including the activating transcription factor 4 (ATF4). ATF4 activates additional downstream effectors, including the pro-apoptotic C/EBP homologous protein (CHOP), which leads to cell death upon prolonged UPR [9]. However, the molecular mechanisms that drive the clinical phenotype of MSS are only partially known. Our research group, in a recent proteomic study of skin fibroblasts from a patient carrying the SIL1 R111X mutation, showed a slight activation of the UPR and an alteration in cell metabolism [10]. In particular, enzymes involved in lipid and amino acid catabolism were upregulated, while those involved in lipid and amino acid synthesis were downregulated, suggesting an increased energy requirement.

A detailed proteomic study of Sil1^Gt^ mouse (murine model of MSS) muscle revealed an increase in chaperones and machinery dedicated to protein degradation already before the onset of clinical muscle pathology [11]. In addition, the phosphatidylinositol-3-kinase (PI3K)/Akt and the mammalian target of rapamycin (mTOR) pathways were activated in conjunction with increased glucose uptake. Finally, a reduction in the expression of important secreted/transmembrane proteins, including the insulin like growth factor 1 (IGF-1) receptor, was found [11]. The alteration of secreted and transmembrane proteins localised in the plasma membrane (PM) account for about 40% of upregulated and 30% of downregulated proteins, an even higher number than the proteins residing in the ER (14% up and 14% down), suggesting that this enormous change could be important for the pathogenesis of MSS [11]. Interestingly, also cytoskeletal proteins (18% up and 10% down) are largely affected in MSS muscle tissue [11]. Our proteomic study confirmed the enrichment of Gene Ontology (GO) terms integrin binding, cell adhesion molecule binding, cell-matrix adhesion mediator activity as well as actin cytoskeleton and actin filament bundle [10]. The huge involvement of cytoskeletal, secreted and PM proteins might indicate a possible key role of extracellular matrix (ECM) changes in MSS pathology. ECM is a network of proteins that provides structural and biochemical support to cells in multicellular organism [12]. It consists mainly of filamentous proteins, including collagen, elastin, fibronectin and laminin, as well as proteoglycans that undergo continuous deposition, degradation and reassembly [12]. This dynamic network of macromolecules influences cell migration, survival and differentiation, thus regulating tissue development. Numerous human diseases affecting different tissues have been associated with alterations in ECM or its receptors, including myopathies and brain diseases [13–17].

In the present study, we performed a transcriptomic analysis on patient-derived fibroblasts to better understand the molecular mechanism of MSS. We identified 664 differentially expressed (DE) transcripts and validated 13 of them by quantitative real-time PCR (qPCR). The majority of DE transcripts belong to intracellular organelles and extracellular space/matrix (ECM) compartments. Functional assays confirmed these changes, showing reduced ECM remodelling capacity, reduced cell motility, and slower spreading during adhesion to Petri dishes. By negative staining transmission electron microscopy (TEM), we revealed that patient fibroblasts readily form huge extracellular fibres not observed in control cells. In vivo, ECM has an important role in tendon tissue, to ensure a functional link between the skeletal muscle fibres and the bone, as well as peri- and intramuscularly, where it contributes in maintaining homeostasis and regulating muscle development. The main component of the ECM in tendons and muscles, collagen, is mainly produced by fibroblasts [18, 19]. ECM components guide the connection between muscle fibres and tendon collagen fibres, regulate the signalling of tendon and muscle progenitor cells, and regulate the maturation of the myotendinous junction (MTJ), where specialised tendon fibroblasts, known as tenocytes, connect to skeletal muscle fibres [20]. To correlate our findings on patient-derived fibroblasts with the woozy mouse model of MSS, we analysed by TEM the ECM produced at the boundary between Achilles tendon and soleus muscle, where a thick band of fibrous connective tissue is present. The structural analysis of the soleus muscle MTJ and surrounding Achilles tendon regions revealed the presence of disorganised collagen fibres in woozy mice, which can in turn significantly impair the mechanical properties of their skeletal muscle.

Our data strongly support the hypothesis that aberrant ECM may be a key pathological feature of MSS and explain most of the clinical manifestations of this syndrome.

## Materials and methods

### Cell cultures

Primary Human Dermal Fibroblast (NDHF Promo Cell #FB60C12350) supplied by Carlo Erba Reagents were used as the control cell line and compared with primary dermal fibroblast from a young Marinesco–Sjögren syndrome patient, supplied by Telethon Network of Genetic Biobanks-TNGB [21]. Cells were cultured in a humidified CO_2_ incubator at a temperature of 37 °C using Dulbecco’s modified Eagle’s medium + GlutaMAX (GIBCO, 61965-026), supplemented with 10% of Fetal Bovine Serum (FBS) and 1% penicillin/streptomycin PenStrep (GIBCO, 15070-063). Cells were subcultured when they reached 90% confluence and detached with Trypsin-EDTA 0.5% (GIBCO, 15400-054). All experiments were performed with cells within the 12th passage.

### Transcriptomic analysis

Total RNA was extracted from control and patient fibroblast in triplicate experiments using the miRNeasy micro kit (Qiagen, 217004) according to the manufacturer’s instructions. Transcriptomic analysis was carried out by next generation sequencing (NGS) RNA sequencing at BMR Genomics Padova, Italy. RNA integrity was determined by analysis of extracted total RNA using a 2100 Bioanalyzer (Agilent Technologies) with RNA 6000 NanoChip. RNA concentrations were measured using Qubit RNA Assay Kit. Libraries were prepared from total RNA according to manufacturer instructions with Lexogen QuantSeq 3’ mRNAseq Fwd kit. Libraries quality were evaluated by size analysis on 2100 Bioanalyzer (Chip DNA HS) and concentrations were determined using Qubit DNA HS assay kit (Thermo Fisher). Sequencing was performed on Illumina Nextseq 500 generating about 5 million/read per sample, SE75 format. Reads preprocessing was performed by using fastp v0.20.0 [22] applying specific parameters in order to remove residual adapter sequences and to keep only high quality data. Passing filter reads were mapped to the genome reference (Homo sapiens) v105 of Ensembl repository using STAR v2.7.0 [23]. Alignments were elaborated by RSEM v1.3.3 [24] and sample-specific gene-level abundances were merged into a single raw expression matrix applying a dedicated RSEM command (rsem-generate-data-matrix). Genes with at least 10 counts in 3 samples were then selected. Differential expression was computed by edgeR [25] from raw counts in each comparison. Multiple testing controlling procedure was applied and genes with a false discovery rate (FDR) ≤ 0.1 and log fold change (FC) > |0.5| were considered differentially expressed. The heatmap represents a selection of the 50 most differentially expressed genes based on the logFC value. In order to create the graph, the R library (pheatmap) with an unbiased hierarchical system was used.

### Computer simulation

In order to understand the significance of the overlap between the 237 biological processes identified in this study and a previous one [10] which identified 898, we generated 898 uniformly distributed pseudo-random integers ranging from 1 to 27,597 (number of biological processes in the GO database) with the Matlab algorithm. These numbers were associated to the biological processes in the GO database. Random generation, association and overlap calculation of these random biological processes with the 237 biological processes identified in this study was repeated 10,000 times. Then, the mean overlap across the 10,000 repetitions was computed.

### Quantitative real-time PCR (q-PCR)

Total RNA from fibroblasts was extracted using TRIzol reagent. RNA was than retrotranscribed using the kit (High Capacity cDNA Reverse Transcription Kit #4368813 from Applied Biosystems) according to the manufacturer’s instructions. The q-RT-PCR reactions, based on SYBER green chemistry (Sensifast SYBR Hi-Rox #BIO-92020 from Bioline), were performed using CFX C1000 (BIORAD) and the expression of selected genes is presented relative to the mean of housekeeping reference, i.e. glyceraldehyde-3-phosphate dehydrogenase (GAPDH) (ΔΔCt method) (Table 1). The PCR primer sequences were as follows:

**Table 1:** Primer sequences used in this study

Gene name	FW sequence 3’-5’	REV sequence 3’-5’
ACAN	CTG CTA TGG AGA CAA GGA TGA G	CTG GAG ATG TTG CAT AAA AGA CC
ALDH1A1	AGC AGG AGT GTT TAC CAA AGA	CCC AGT TCT CTT CCA TTT CCA G
CTHRC1	CAA TGG CAT TCC GGG TAC AC	GTA CAC TCC GCA ATT TTC CCA A
CTTNBP2	CCG TGG GAC TTT ATG AGG AAG	GAG GGA TCA TGG AGG CAT AAG
ELAPOR2	CCT GGC AAC ATG AAA ACT TCC	TCA TTG TCA GAA CCT CCA GC
EXPH5	TGG GAC AGA AAG GAT GTG ATG	CTG GCT TTG GGA CAA ATG TAC
GAPDH	CTG GGC TAC ACT GAG CAC C	AAG TGG TCG TTG AGG GCA ATG
GPC4	AGG AAA CGG CAA TGA GGA TG	GAC GAA GGA TCA GTA TGT CTG G
LXN	ACA GAA CTA CAT CAA CTA CCA GC	GTG ATA CTT ATG TCC TCT TCC TGG
NRN1	ACA AGA CGA ACA TCA AGA CCG	TTA TCC CAC ATA TCT TTC GCC C
PCDH7	TGT CAA ACC AAT AAC AAG TAC AGC	ACT GCT TGG TGT TTC TGA CTC
PCOLCE2	GGG AAG TCA ACG ATG CTA GAA G	GTG ACC AAT AAA CCC ATC TGC
SIL1	GCT TCA CCT TCT GCC TCA GTC A	AAC ACC TCC AGG ACT TCG GCA T
VCAN	GGT GGT CTA CTT GGG GTG AG	GTG ATG CAG TTT CTG CGA GGA

One-way ANOVAs were performed to compare ΔCt values for each gene between the two groups. Then Benjamini-Hochberg adjusted p-values were computed to adjust for multiple comparisons.

### Collagen degradation/internalization assay

Control and patient fibroblasts were seeded in 24-well plates (3 × 10^4^ cell/well) or into ibidi µ-slide 8-well glass-bottom plates (19,5 × 10^2^ cell/well) for widefield or confocal analysis, under standard growth conditions. After 24 h medium was replaced with Dulbecco’s Modified Eagle Medium (DMEM) serum‐free and after 16 h cells were incubated with 10 µg/mL of dye-quenched (DQ)-collagen (Invitrogen, Carlsbad, CA) up to 5 h. Collagen degradation was monitored by detection of the green signal in a fluorescence microscope (EVOS^®^FL, AMG, USA). The fluorescence intensity of each cell was calculated using a formula for corrected total cell fluorescence (CTCF) = integrated density–(area of selected cell × mean fluorescence of background readings) [26]. For confocal analysis, cells were treated as above and exposed to LysoTracker (ThermoFisher) as for the manufacturer’s instructions. Images were acquired with a Zeiss LSM880 confocal microscope (Zeiss) using 495 and 647‐nm lasers and as previously described [27]. Quantification of DQ-collagen internalization in confocal images was performed according to the procedure described above for the fluorescence microscope and expressed as CTCF.

### Collagenase assays

Cell-free supernatants were tested for collagenase activity with the EnzChek Gelatinase/Collagenase assay kit (Invitrogen, Carlsbad, CA). Control and patient fibroblasts were seeded in 24-well plates 3 × 10^4^ cell/well under standard growth conditions, and after 24 h medium was replaced with DMEM serum‐free. After 16 h cell supernatants were collected, centrifugated at 3000 g for 10 min at room temperature, and filtered using a 0.2 μm Minisart sterile filter (Sartorius). Reactions were prepared by mixing 100 µL of conditioned media with 20 µL of the substrate at 100 µg/mL and 80 µL of reaction buffer in black optical bottom 96- well plates (Greiner Bio-One, Monroe, NC). Plates were incubated in a Synergy H1 microplate reader (BioTek, Winooski, VT) at 37 °C in atmospheric conditions. Kinetic fluorescence reads were measured after 7 s shaking every 5 min at 485 nm excitation/527 nm emission. There was no need to normalize collagenase activity because the total amount of proteins, at the time of the experiment, was the same in the control and in the patient fibroblasts.

### Cell adhesion assay

Control and patient fibroblasts were seeded in 96-well plates (5 × 10^2^ cells/well) and settled at RT for 15 min before being placed into Incucyte^®^ Live Cell Analysis System (Incucyte^®^ S3/ -Sartorius) at 37 °C in a humidified CO2 incubator. Four phase-contrast images of each well were acquired every 3 h for 24 h using 10X objective. Images were analysed by Incucyte^®^ Cell-by-Cell Analysis Software, and data were exported for further statistical analysis on GraphPad Prism 9. Statistical differences in the average phase object area between control and patient fibroblasts over time were determined using repeated measure ANOVA over the 9 time points. Fisher LSD post-hoc test was used to compare average phase object area value at the same time point between groups. P value < 0.05 was the threshold for statistical significance.

### Motility assay

Cell motility was assessed according to the protocol for Incucyte Scratch Wound Assay. Control and patient fibroblasts were seeded in 96-well ImageLock™ plate to reach maximum density of 100% (2 × 10^4^ cells/well) and incubated overnight in standard growth conditions. The next day, the 96-pin Incucyte WoundMaker (Sartorius) was used to create a precise and uniform wound simultaneously in each well. Cells were washed twice with PBS and then incubated into Incucyte^®^ Live Cell Analysis System (Incucyte^®^ S3/ -Sartorius). Two phase contrast images from each well were acquired every 3 h for 24 h using 10X objective. Relative wound density was analysed using Incucyte^®^ Scratch Wound Analysis Software Module, and data were exported for further statistical analysis on GraphPad Prism. Statistical differences in the relative wound density between control and patient fibroblasts over time were determined using repeated measure ANOVA as described for the average phase object area. P value < 0.05 was the threshold for statistical significance.

### Transmission electron microscopy (TEM) of soluble ECM

Controls and patient fibroblasts at 80% of confluency were incubated with serum and antibiotic-free medium over-night. The next day, the conditioned medium was collected and centrifuged at 20,000 rpm to remove any residual cells or cellular debris. The protein concentration of the conditioned medium quantified by was on average 14.0 ± 1.8 and 10.8 ± 1.0 µg/mL for control and patient fibroblasts, respectively.

The overall morphology of the ECM samples collected as described, has been characterized by TEM using a CM 100 transmission electron microscope (Philips) equipped with a tungsten filament and operating at 100 kV. The specimens have been prepared by negative staining as previously described for protein molecules [28]. Briefly, a 5 µL drop of each ECM sample at concentration 10–15 µg/mL has been applied onto a 200-mesh carbon-coated grid (Agar Scientific Ltd) and let adsorb 1 min before dropping it downwards on 100 µL commercial Nano-W stain (2% organotungsten compound, Nanoprobes Inc.). Thereafter, the excess of stain has been removed by touching the grid side with Whatman filter paper (Merck KGaA) and the grid left to air-dry before imaging by TEM. When necessary, the acquired micrographs have been post-processed using a high-pass or fast Fourier transform (FFT) bandpass filter by means of ImageJ v1.54 to reduce edge artifacts derived from low or over-exposure to the electron beam.

### Mice

Woozy mice (CXB5/By-Sil1wz/J) purchased from The Jackson Laboratory (Stock No. 003777), housed at controlled humidity and temperature were provided with a standard diet and water ad libitum. The mouse colony was propagated by heterozygous mating and genotyped by standard PCR. All procedures involving the mice were performed according to the protocol approved by the Institutional Animal Care and Use Committee of the Italian Ministry of Health (authorization n° 596/2021-PR, issued pursuant to art. 31 of Legislative Decree 26/2014). Animal housing facilities meet international standards and are regularly inspected by a certified veterinarian.

### Histology and TEM of woozy mouse Achilles tendon

Samples were carefully dissected from 7 months old woozy (*n* = 3) and control (*n* = 3) mice, pinned on Sylgard dishes, fixed at room temperature with 3.5% glutaraldehyde in 0.1 M NaCaCO buffer (pH 7.2) and kept at + 4 °C in fixative solution until further use. Fixed samples were then postfixed in 2% OsO_4_ in the same buffer for 2 h, then block-stained in uranyl acetate replacement. After dehydration, specimens were embedded in an epoxy resin (Epon 812; Electron Microscopy Sciences, Hatfield, PA, USA), as previously described [29, 30]. For histological examination by light microscopy, semithin sections (∼700 nm) were cut using a Leica Ultracut R microtome (Leica Microsystem, Vienna, Austria) with a diamond knife (Diatome, Biel, Switzerland) and stained in a solution containing 1% toluidine blueO and 1% sodium borate (tetra) in distilled water for 3 min on a hot plate at 55–60 °C. After washing and drying, sections were mounted with DPX media for histology (Sigma–Aldrich, Milan, Italy) and observed with a Leica DMLB light microscope connected to a DFC450 camera equipped with Application Suite v 4.13.0 for Windows (Leica Microsystem, CMS GmbH, Switzerland). For TEM, ultrathin sections (∼50 nm) were cut using a Leica Ultracut R microtome (Leica Microsystem, Vienna, Austria) with a Diatome diamond knife (Diatome, Biel, Switzerland) and double-stained with uranyl acetate replacement and lead citrate. Sections were viewed in a in a 120 kV JEM-1400 Flash Transmission Electron Microscope (Jeol Ltd, Tokyo, Japan) equipped with CMOS camera Matataki and SightX Viewer software (Jeol Ltd, Tokyo, Japan) at 80 kV.

## Results

### Changes in the extracellular matrix and anchoring junctions in fibroblast isolated from a patient affected by Marinesco-Sjögren syndrome

**Fig. 1:**
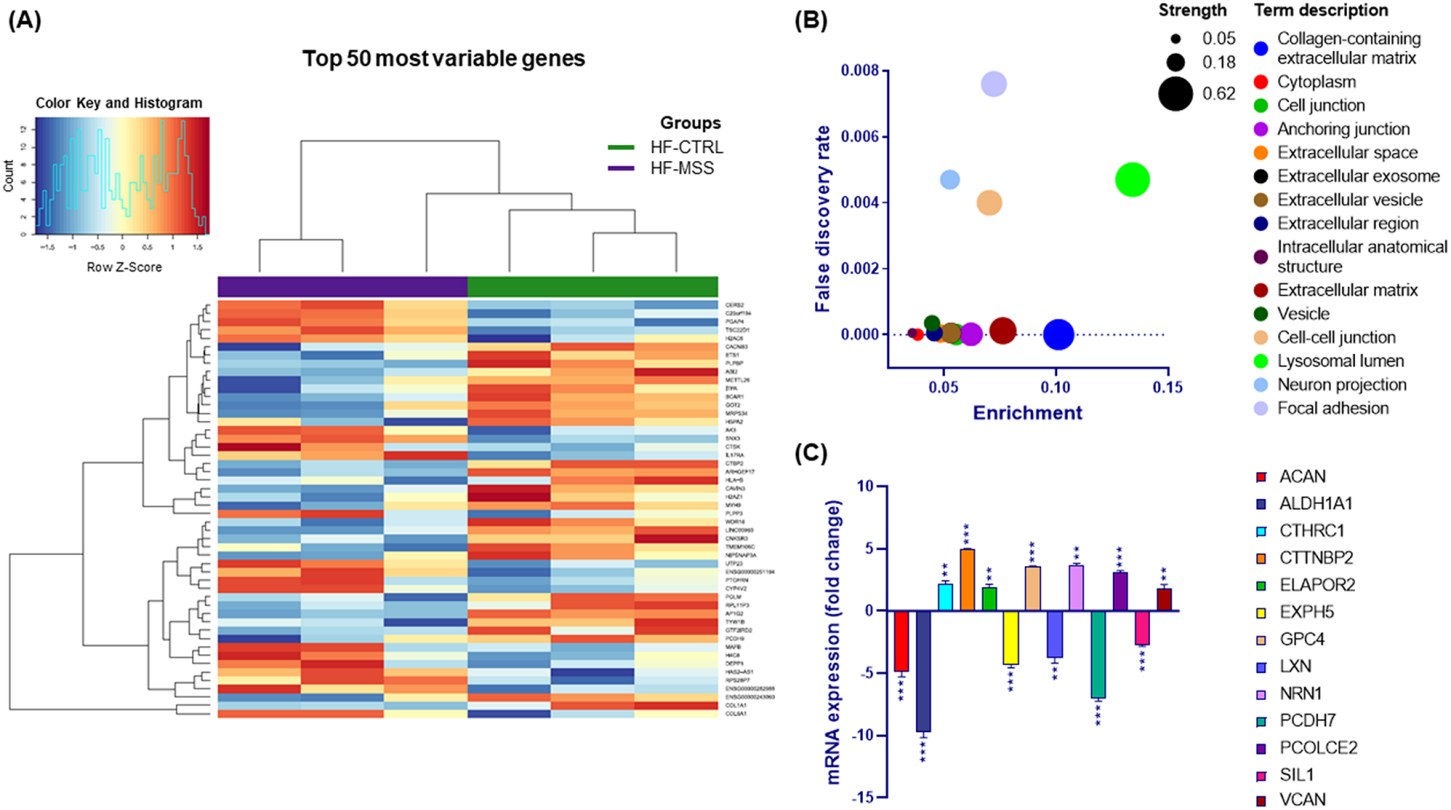
Differentially expressed genes in fibroblasts from an MSS patient compared with a healthy control. **A**) Heat map of the modulated genes. The heatmap shows the top 50 most variable genes based on the logFC value. Upregulated genes are reddish while downregulated genes are in blueish as reported in the colour key histogram. Control and patient fibroblast samples are indicated by a green and violet bar respectively. Gene names are indicated on the right. (**B**) Bubble plot of cellular components enriched by differentially expressed genes. Enrichment is calculated as the ratio of the number of observed genes to the total number of genes associated with that specific ontology. The strength represents the Log10 of the ratio between the number of genes annotated to a specific ontology and the number of genes expected to be annotated to that ontology if they were picked randomly from a group of the same size. (**C**) Quantitative PCR analysis. Twelve differentially expressed genes identified by transcriptomic analysis were validated by quantitative reverse transcriptase PCR. Data are expressed as fold change and gene names are in the legend shown on the right. One-way ANOVA was performed to compare ΔCt values. ** *p* > 0.01, *** *p* > 0.001. Results are presented as mean ± standard error of fold changes from three independent experiments performed in technical triplicate

To shed light on the pathogenic mechanism of MSS we performed a transcriptomic analysis of skin fibroblasts isolated from a young patient affected by this disease. Total RNA extracted from fibroblasts, cultured under standard conditions, was used to assess gene expression by outsourced next-generation sequencing. As a control, fibroblasts from a healthy individual were treated in parallel. The experiments performed in three biological replicates identified 664 differentially expressed (DE) transcripts having a fold change (FC) > of 2 and FDR < 0.1 (Supplementary Table 1). The top 50 most variable genes, based on unbiased hierarchical clustering of the logFC value, are shown in the heat map (Fig. 1A). Analysis of the relationships between DE transcripts using STRING protein-protein interaction database indicated that these transcripts have significantly more interactions than if 631 genes (those present in STRING database) had been chosen at random (*p* < 6.7 10^− 12^). This supports the reliability of the transcriptomic results.

In order to gain insight about the functional effects of such changes in the transcriptome we searched for possible enrichment of DE genes with respect to gene ontology (GO) classification. Overall, our gene set resulted to be enriched for 237 different GO biological processes, 6 molecular functions, 30 cellular components and 24 subcellular compartments (Supplementary Tables 2, 3, 4 and 5). As expected by previous studies, enrichment analysis confirmed that the patient fibroblasts have a membrane trafficking issue. In fact, intracellular organelles account for 4 out of 24 subcellular compartments and are representative of more than 350 DE genes (Supplementary Table 5). Interestingly, the most represented subcellular compartments are the extracellular space/matrix and those dedicated to cell-matrix and cell-cell adhesion. Altogether, these account for 15 of the 24 compartments to which more than 150 DE genes belong (Supplementary Table 5). The involvement of both the endomembrane and the ECM/cell adhesion issue was confirmed by looking at the enrichment of the cellular components (Fig. 1B and Supplementary Table 4).

To infer the extent to which the gene alteration identified in this study is representative of MSS, we compared the enriched biological processes (Supplementary Table 2) with those of our previous proteomic study on the same fibroblasts [10] and with those of Sil1^Gt^ mouse muscle [11]. To make this comparison, we first used the STRING database to identify biological processes enriched in the DE proteins reported in the proteomic study of Sil1^Gt^ mouse muscle (Supplementary Table 6). About 50% of the biological processes were shared between the current transcriptomics and our previous proteomics study performed on the same cells [10]; while the overlap with mouse muscle proteomics (Supplementary Table 6) was about 30%. To understand the significance of this degree of overlap, we performed a computer simulation. Indeed, 898 randomly selected biological processes (see methods), i.e. the number of biological processes identified in our previous proteomic study [10], have only 3.2% overlap with those reported in Supplementary Table 2. On this basis, we conclude that the degree of overlap of the biological processes identified in this study with previous ones (around 50%and 30%) is highly significant and far from what could be obtained by chance (around 3%). This suggests that our transcriptomic profiling has identified important molecular pathology clues shared by this syndrome across species and tissues.

Next, we used the web-based software Ingenuity Pathways Analysis (IPA) to deepen the functional analysis of DE genes. An analysis of “Canonical Pathways” (well established and manually curated metabolic and signalling pathways) potentially influenced by DE genes identified glycoprotein VI (GP6) signalling and glioma invasiveness signalling as significantly downregulated (Z-score < 2) in patient fibroblasts compared to controls. However, several other ECM-related pathways have been downregulated, including the tumour microenvironment, the signalling by Rho family GTPases, the pulmonary fibrosis idiopathic signalling and the hepatic fibrosis signalling.

Subsequently, using the IPA module called “Disease and Functions” we identified cellular movement of tumour cell line and migration of tumour cell line as significantly decreased (Z-score < 2). Furthermore, apoptosis was increased (Z-score 3.03) while cell survival was decreased (Z-score − 2.38). Finally, cell proliferation of tumour cell lines was also decreased (Z-score − 2.25). This analysis confirms the involvement of the ECM and indicates a potential increase in cell death and inhibition of cell proliferation, as reported in our previous study [10].

To experimentally assess the reliability of our transcriptomic analysis, 13 DE genes, including SIL1 itself, were validated by quantitative real-time PCR (qPCR). The genes to be validated were chosen among those with higher fold changes and lower FDR. However, with the aim of covering the different functional alterations associated with MSS we included genes belonging to the ECM, cell cytoskeleton, vesicle trafficking and endosome-lysosome-autophagy. Total RNA was extracted from controls and patient fibroblasts, reverse transcribed, and used for qPCR analysis. The qPCR confirmed the upregulation of seven genes and the downregulation of five genes (Fig. 1C).

### Remodelling of extracellular matrix is impaired in patient fibroblasts

As the observed gene changes could be involved in the formation/organisation of the ECM and in anchoring junctions, we studied ECM remodelling to assess whether the observed molecular changes could be reflected in a functional phenotype. Cells remodel the ECM by means of metalloproteases (e.g. collagenase). The cleaved matrix proteins can then be internalised and eventually hydrolysed in the endo-lysosomal compartment to recycle the components. To monitor this phenomenon, we evaluated the digestion and uptake of a molecular probe consisting of a fluorescein-labelled, highly quenched collagen (dye-quenched (DQ) -collagen). DQ-collagen can only fluoresce after digestion by collagenases. The digested probe is internalised by the cells and can be visualised by immunofluorescence microscopy.

**Fig. 2:**
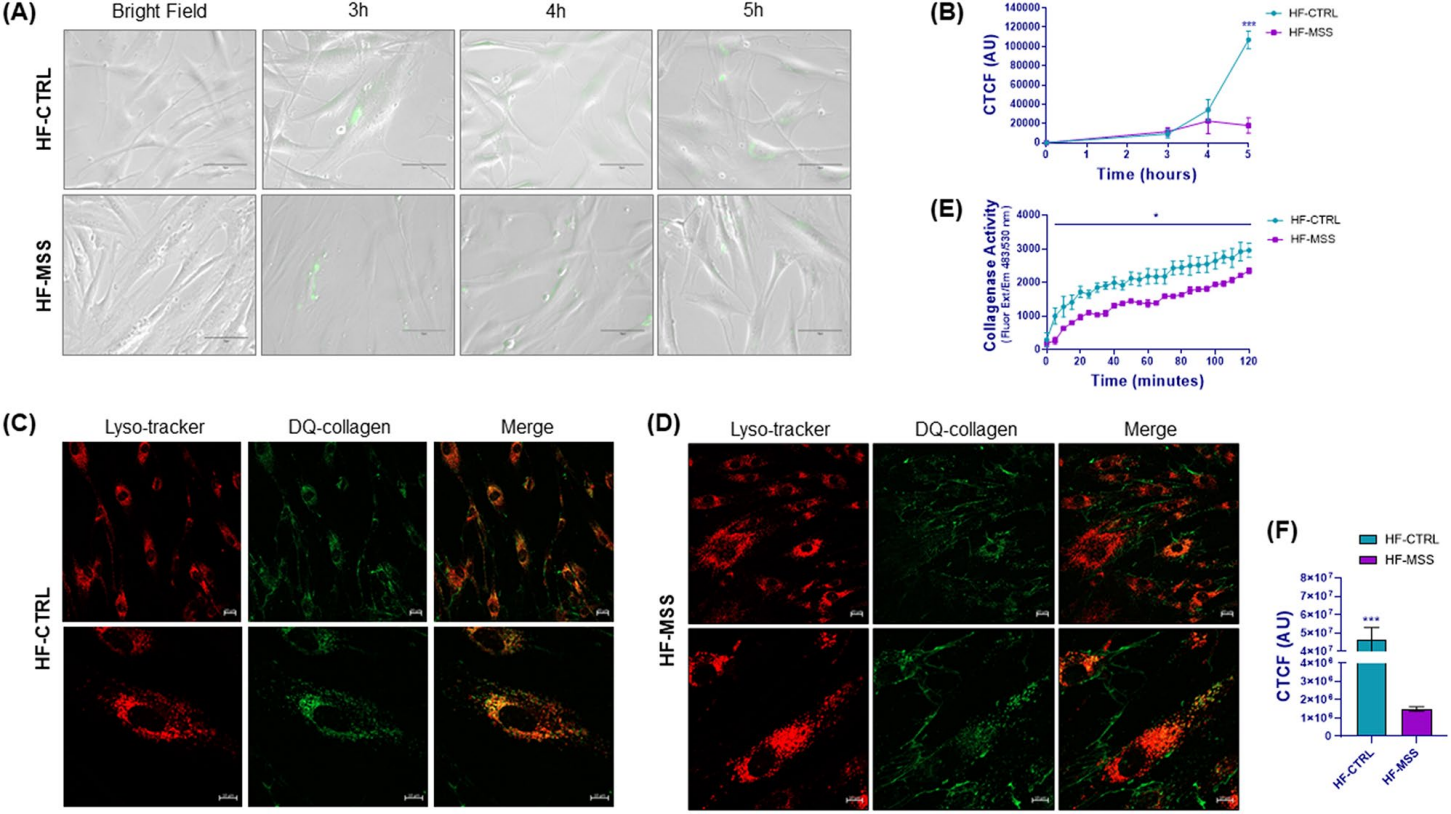
Live cell imaging of DQ-collagen internalization in fibroblasts and collagenase activity. (**A**) Panels show representative images of DQ-collagen fluorescence internalised in control (HF-CTRL) and patient fibroblasts (HF-MSS) over time. (**B**) Quantitation of the fluorescence of the experiment shown in A. The y axis represents the corrected total cell fluorescence (CTCF) expressed as arbitrary units. Results are presented as mean ± standard error from three independent experiments. Repeated measure ANOVA over time points and post parametric multiple t test were performed; *** *p* < 0.001q < 0.001. (**C**-**D**) Control fibroblasts (HF-CTRL in C) and patient fibroblasts (HF-MSS in D) grown on ibidi µ-slide were incubated with DQ-collagen for 5 h, followed by LysoTracker for 3 h. Live cells were imaged by confocal microscope using the 495 and 647-nm lasers. LysoTracker and DQ-collagen fluorescence are shown in red and green respectively. Colocalization of lysosomes and DQ-collagen is shown by merging the fluorescence. Two different magnifications are shown. Results are from two independent experiments performed in technical triplicate. (**E**) Collagenase activity of fibroblast conditioned medium. Cell-free supernatants of fibroblasts were incubated with DQ-collagen and fluorescence development was measured every 5 min. Results are presented as mean ± standard error from two independent experiments performed in technical triplicate. Repeated measure ANOVA over time points and post parametric multiple t test were performed; * *P* < 0.05. (**F**) The graph shows the quantification of internalized collagen in HF-CTRL and HF-MSS of the experiments shown in C and D. *** *P* < 0.001

Time course experiments showed that at 3 h of incubation with DQ-collagen, fluorescent dots appeared in both control and patient fibroblasts (Fig. 2A). However, at longer times, fluorescence increased further only in control cells, while it remained low in patient cells (Fig. 2A and B). We believe that in patient cells, reduced expression of metalloproteases and increased expression of metalloprotease inhibitors (Supplementary Table 1) impair ECM remodelling, therefore DQ-collagen is eventually processed and internalized to a lesser extent.

Colocalization experiments using confocal microscopy showed that intracellular dots colocalise with the LysoTracker, used as a marker for lysosomes (Fig. 2C and D). On average, the amount of fluorescent probe internalised by patients cells was very little in comparison to control fibroblasts (Fig. 2C, D and F). In addition, fluorescent filaments surrounding the cells were detected in the cultures; these were most prominent in patient fibroblasts (Fig. 2C and D and Supplementary Fig. 7), suggesting a slowing of ECM metabolism in diseased cells. To clarify whether this scenario was due to reduced metalloprotease activity, we measured the ability of the fibroblast conditioned medium to digest DQ-collagen and emit fluorescence. Indeed, the proteolytic activity measured in the conditioned medium collected from patient fibroblasts was lower than that measured in the conditioned medium from control fibroblasts (Fig. 2E).

These data support the idea that the molecular changes identified by transcriptomics, impact ECM remodelling.

### Transmission electron microscope analysis showed the presence of proteinaceous fibres only in the ECM produced by the patient fibroblasts

**Fig. 3:**
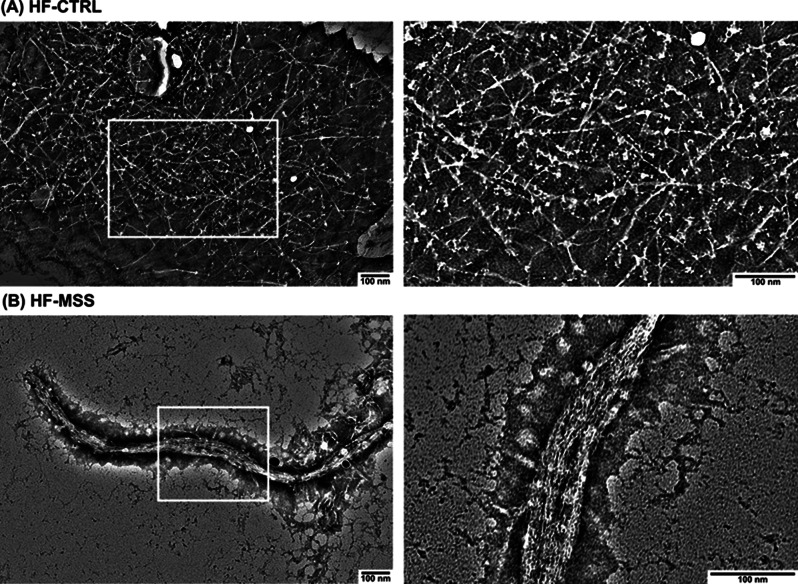
TEM analysis of negative-stained ECM released by fibroblasts. Fibroblasts grown to 80% of confluence were incubated with serum free HBSS medium for 24 h. The day after, the conditioned medium was collected, refrigerated and a 5 µL drop has been applied onto carbon-coated grid. The grid was stained and air-dried before imaging by TEM. (**A**) Representative micrographs of ECM generated by control fibroblast (HF-CTRL), at low (left) and high (right) magnification. (**B**) Representative micrographs of ECM generated by patient fibroblast (HF-MSS), at low (left) and high (right) magnification. Results are from two independent experiments performed in technical triplicate

In order to directly observe the consequences of a possible alteration of ECM processing, we collected the conditioned medium of fibroblasts and analysed its organisation by negative-staining TEM. Electron micrographs of the conditioned medium of control fibroblasts showed thin protein filaments with similar thickness and shape likely organized as a network (Fig. 3A). Accounting for the size of these filaments, the average estimated value of the thickness was 11 ± 1.8 nm. Likewise, the conditioned medium of the patient fibroblasts showed an overall network organisation, but, interestingly, the filaments were thicker and exhibited an apparent ribbon-like shape (Fig. 3B). Furthermore, this morphology likely reveals the tendency of the filaments to aggregate and form a sort of primitive fibrils, (Fig. 3B), that are reminiscent of collagen organization [31]. As a result, the calculated thickness of these aggregates increased up to 48.8 ± 16.2 nm on average, thus confirming increased size while pointing out to large variability throughout the sample. Note that these overstructured filaments in fibrillar form (Fig. 3B right panel) were not detected in the conditioned medium of the control fibroblasts. No further molecular differences between the control and patient-derived filaments can be observed due to the resolution limit of the microscope.

These data suggest that the ECM of healthy fibroblasts is still immature at 24 h of deposition, most probably because the formation of large fibrils requires longer remodelling times. Whereas the diseased cells, which show altered remodelling activity (proteolytic and/or cross-linking activity), at 24 h have formed larger but probably disorganized fibrillar aggregates.

### The change in cell morphology during attachment to Petri dishes and cell motility are slowed down in patient fibroblasts

Prompted by the numerous changes in the expression of genes belonging to the ECM, cytoskeleton and anchor junctions, as well as by evidences of an abnormal ECM, we decided to analyse the rapidity of adhesion to Petri dishes and cell motility.

**Fig. 4:**
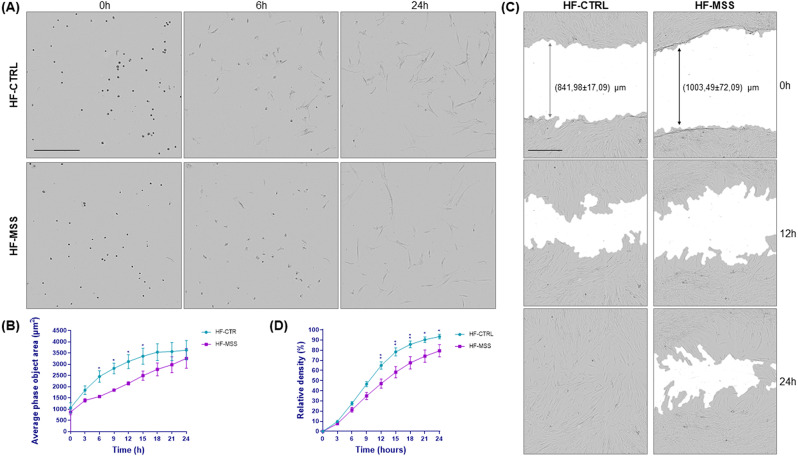
Analysis of fibroblast-ECM interactions. (**A**) Fibroblast attachment rate. Control (HF-CTRL) and patient fibroblasts (HF-MSS) were plated on a 96 well plate and imaged immediately after and then every three hours for 24 h. Images are shown at 0, 6 and 24 h of incubation as indicated. Differences in attachment rate are already visible after 6 h. Scale bar is 400 μm. (**B**) Graph shows the area occupied by the cells over time. Results are presented as mean ± standard error from three independent experiments performed in technical quadruplicate. Repeated measure ANOVA over time points and post parametric multiple t test were performed; * *p* < 0.05. (**C**) Wound-healing assay. Control (HF-CTRL) and patient fibroblasts (HF-MSS) plated at confluency on a 96 well plate were scratched and wound closure was imaged every three hours for 24 h. Images show differences in cell migration at 0 h, 12 h and 24 h of incubation. Scale bar is 400 μm (**D**) Quantitative analysis of the experiment shown in B. Cell migration is expressed as relative wound density. Results are presented as mean ± standard error from four independent experiments performed in technical quadruplicate. Repeated measure ANOVA over time points and post parametric multiple t test were performed; * *p* < 0.05, **p < 0.01

To assess how quickly cells enlarge in size during attachment to the Petri dishes, we monitored the area occupied by the cells immediately after plating, when they are mostly round, and at 3 h intervals up to 24 h, when the cells can be considered fully enlarged on the dish, as their area does not increase further over time. This assay is indicative of cell-ECM interaction and cytoskeleton dynamics. Interestingly, patient cells reached full extension more slowly than controls (Fig. 4A and B), probably due to the fibrillar supramolecular assembly of the aberrant ECM matrix, as observed by confocal microscopy and TEM (Figs. 2 and 3), as well as to altered expression of adhesion receptors.

Cell motility was assessed by wound-healing and analysed by Scratch Wound Analysis Software (Sartorius). Cells were plated to confluency, scratched and wound closure was monitored at 3 h intervals for 24 h. It should be noted that the Sartorius 96-well wound-maker reproducibly generated scratch widths of approximately 800 μm in controls and 1000 μm in patient fibroblasts (Fig. 4C). This difference cannot be explained by the larger size of the patient cells [10], but probably depends on the removal of additional rows of cells in addition to those directly removed by the cutting blade, because the diseased cells are more strongly attached to each other. Cell motility, calculated by measuring the relative density of the wound in the scratched area compared to the confluent area, showed slower movement of diseased fibroblasts compared to controls (Fig. 4D).

However, we would like to emphasize that we cannot exclude that intrinsic cellular factors (e.g., cellular stress), in addition to alteration of the ECM, may contribute to slow cell attachment and motility.

### Aberrant ECM deposition/remodelling impact tendon thickness/organization in woozy mouse

On the basis of the striking phenotype observed in the ECM generated by fibroblasts in culture, we wondered whether altered ECM deposition/remodelling could also affect the woozy mouse model of MSS. To this aim, the boundary between Achilles tendon and the soleus muscle from seven months old woozy and control mice were fixed and analysed by TEM.

Adult tendon is a collagen-rich tissue, populated with a small number of flat specialized fibroblasts called tenocytes [32]. In the ECM, collagen is organized in fibrils with a regular striation, and collagen fibrils are disposed in bundles of fibres. Embedded in the ECM of the tendon are the tapered ends of skeletal muscle fibres, which form the MTJ. Skeletal muscle fibres develop extensive folding of sarcolemma at this interface and tendon extensions are elongated and branched throughout the MTJ interface.

**Fig. 5:**
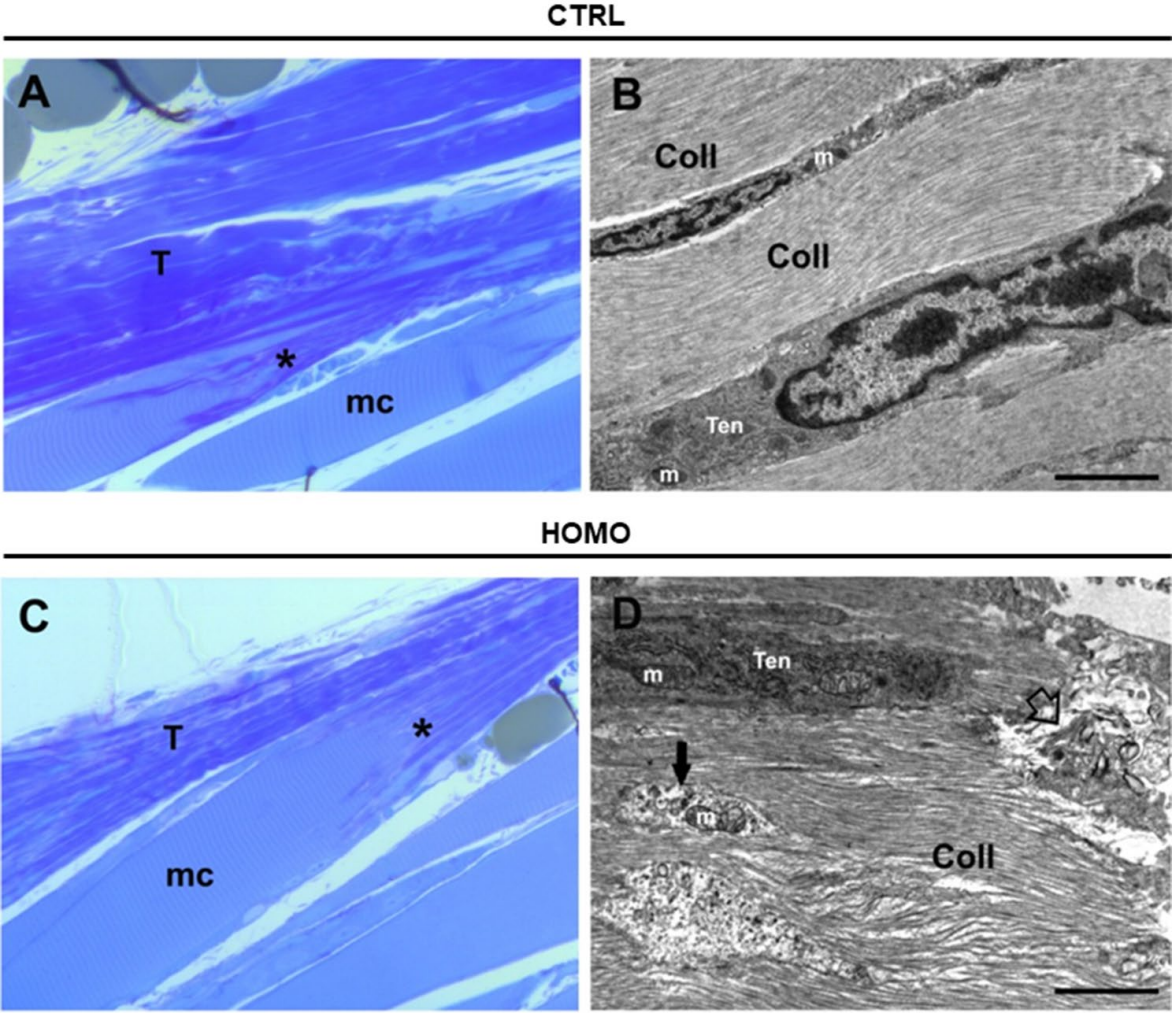
Light and transmission electron microscopy (TEM) analysis of Achilles tendons of control and woozy mouse. (**A** and **C**) Toluidine blue stained semi thin-sections of the boundary between Achilles tendon (T) and the soleus muscle (mc) from heterozygous (*SIL1*^+/−^) control (CTRL, A) and woozy (*SIL1*^−/−^) mice (HOMO, **C**). The myotendinous junction (MTJ) is indicated by asterisk. (**B** and **D**) Representative images at TEM higher magnification of the connective tissue in control (CTRL, B) and woozy mice (HOMO, D), with tenocytes (Ten) arranged between the collagenous fibrils (Coll) of the ECM. Some organelles are visible (e.g. mitochondria, m) as well as cell debris (black arrow), and areas of degeneration (empty arrow)

In semi-thin sections stained with toluidine blue, collagenous fibres at the boundary between Achilles tendon and the soleus muscle were well identified as dark blue stained regions in control animals, and a well distinct striation pattern was present (Fig. 5A and B). In woozy mice samples extensive changes in the organization of connective tissue arrangement was observed (Fig. 5C and D). In the tendon matrix there is less organized fibrous connective tissue, and collagen fibres appear less compact as demonstrated by the irregular blue stain (Fig. 5C). The MTJ regions in each group were identified (asterisks in Fig. 5A and C).

**Fig. 6:**
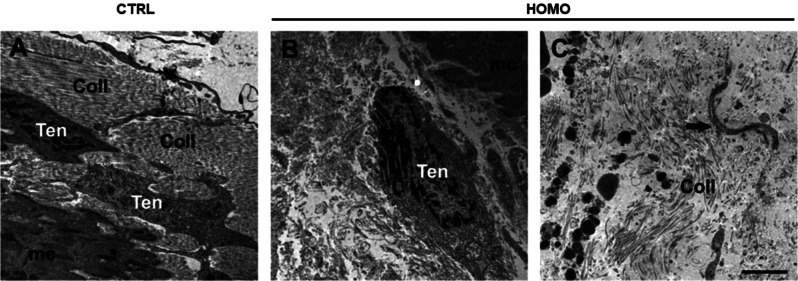
TEM analysis of the MTJ region in control and woozy mice. **A** -**C**) Ultrastructural appearance of the muscle (mc)/tendon interface in heterozygous (*SIL1*^+/−^) control (CTRL, A) and woozy (*SIL1*^−/−^) mice (HOMO, B). Tenocytes (Ten) derived from the tendon tissue adjacent to the MTJ are surrounded by collagen fibrils (Coll). Sometimes in the ECM overstructured filaments in fibrillar form are present (black arrow in C). Scale bar 2 μm

Analysis at TEM higher magnifications, of the muscle/tendon interface confirmed our observations in ultra-thin sections. Well-aligned collagen fibres are visible in control samples (Fig. 5B), compared to a more irregular pattern in samples from woozy mice (Fig. 5D). The presence of tenocytes was observed in the electron micrographs of both groups of mice. In longitudinal sections the tenocytes of control animals often have an oblong shape with oval nucleus and are arranged between the collagenous fibrils of the ECM (Fig. 5B and D). In cross-section (images not shown) they present the typical triangular irregular shape of tenocytes, sometimes also visible in longitudinal section (Fig. 6A), with long finger-like cellular processes that extend into the ECM and attach to the collagenous fibrils. The cell organelles of the tenocytes appear intact, including the rough ER and the mitochondria, which present a regular appearance (Fig. 5B). In samples from woozy mice, tenocytes derived from the tendon tissue adjacent to the MTJ have degenerative alterations, such as damaged mitochondria, and cell debris are often found in the nearby ECM ( Fig. 5D). Tenocytes are surrounded by collagen fibres often lacking a well-organized pattern of disposition (Fig. 6B). Sometimes tenocytes have rounder shape, with a large nucleus and fewer cellular processes and are surrounded by collagen fragments and/or various material, not always well identifiable, sometimes resembling overstructured filaments in fibrillar form found in the conditioned medium of the patient fibroblasts (Figs. 3B and 6C).

## Discussion

MSS is an early-onset autosomal recessive genetic disorder that manifests with different degrees of severity; typically there is ataxia and myopathy, some degree of mental retardation that can range from mild to severe, and widely varying skeletal and eye problems [1–3, 33, 34]. This phenotype is triggered, at least in about 60% of the cases, by loss of functions mutations of Sil1, a ubiquitous cochaperone resident in the ER. However, so far the clinical variability has not been linked to specific Sil1 mutations, but rather could be attributed to the patient’s genetic background. Sil1 loss impairs protein folding in the ER and consequently UPR is generated. The UPR is probably involved in the pathogenesis of MSS, although the exact molecular mechanisms that (i) cause the selective degeneration of Purkinje neurons and muscle fibres, (ii) have important consequences on cortical neurons (mental retardation) and chondrocytes (short stature) and (iii) spare many other cells such as leukocytes or fibroblasts are unknown [2].

We believe that identifying the molecular mechanisms underlying the selective degeneration of cells and/or the mechanisms activated by cells that are able to cope with the loss of Sil1 (e.g. fibroblasts) is crucial to fully understanding this syndrome and may pave the way for therapy. For this purpose, we analysed the gene expression of fibroblasts isolated from a patient and compared it with a healthy control. This patient carries a nonsense DNA mutation in the SIL1 gene that generates the R111X alteration on the protein [10]. This truncated Sil1 is probably degraded because it is undetectable by western blotting (data not shown). Patient-derived fibroblasts showed more than 600 transcripts with altered expression. The expression of a few DE transcripts was confirmed by qPCR using total RNA extracted from the same fibroblasts. In order to assess the general reliability of the transcriptomics we showed, by statistical analysis, that the DE transcripts are correlated with each other and are not the result of random selection. Furthermore, the enriched biological processes linked to DE transcripts significantly overlap with those previously identified by proteomic analysis in the same fibroblast [10] and muscle of a mouse model of MSS [35], thus supporting the predictive value of this transcriptomics for MSS.

Bioinformatic analysis of DE transcripts showed that gene expression changes are mainly associated with intracellular organelles, including lysosomes. This result is in line with previous studies showing that intracellular organelles and autophagic vacuoles are the main subcellular compartment affected by MSS [10, 36, 37]. Our analysis also revealed possible alterations in ECM formation and remodelling based on changes in collagens, glycosaminoglycans, metalloproteases and metalloprotease inhibitors (Supplementary Table 1). Complementing this, the expression of anchoring junctions genes, such as integrins, and cytoskeleton genes, including filamin, alpha-actinin-4 and the neural cell adhesion molecule L1, was also affected. However, it is difficult to say whether the alterations in the expression of transcripts belonging to the anchoring junctions and the cytoskeleton are primitive or secondary to changes in the ECM.

**Fig. 7:**
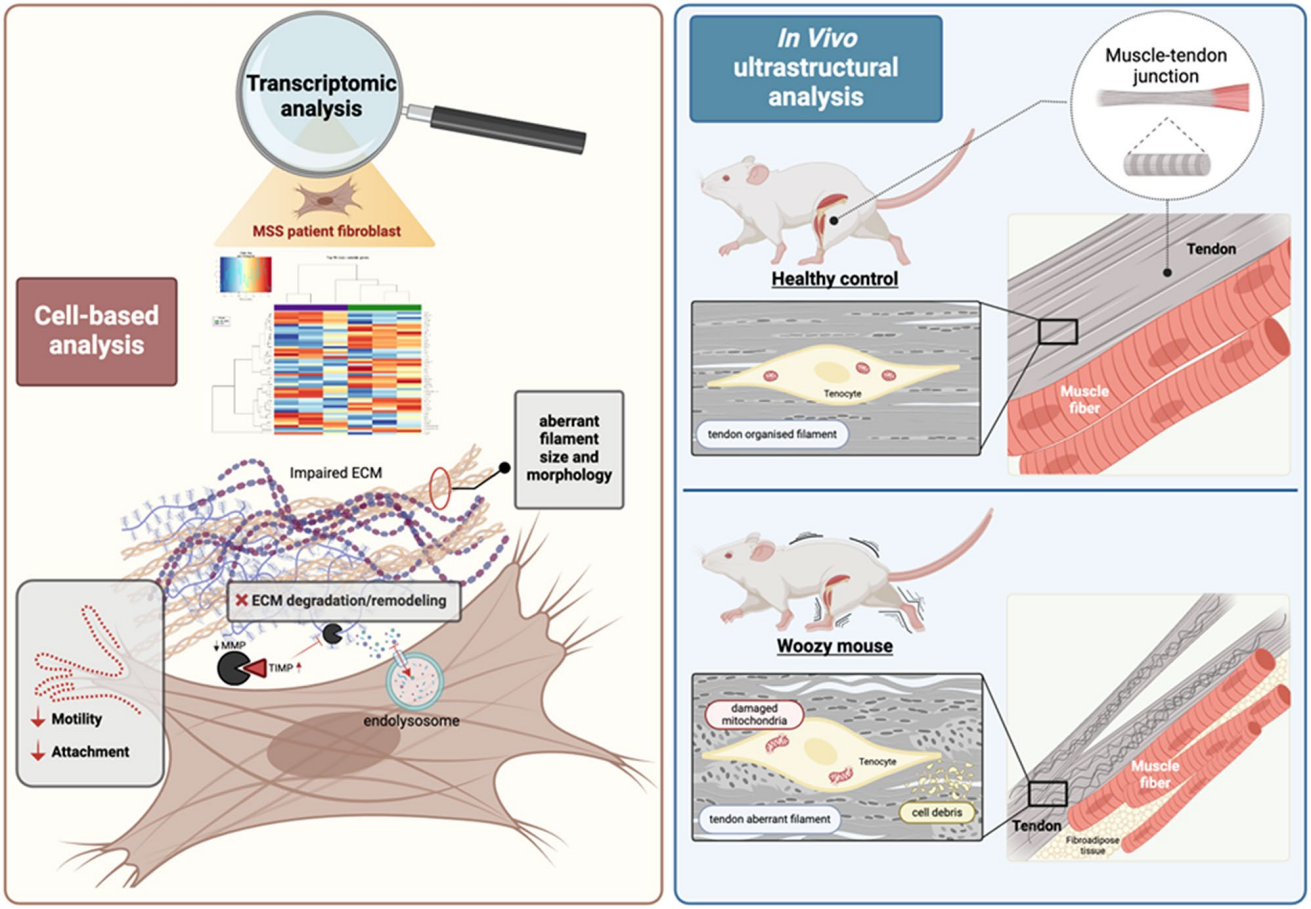
Through molecular, cellular and histological approaches in vitro and in vivo, we revealed changes in the ECM and tendons of woozy mice that may explain part of the pathological mechanism of MSS. The left panel summarises in vitro experiments showing genes differentially expressed in control and patient fibroblasts. These molecular changes impair ECM dynamics, resulting in an aberrant ECM that in turn slows cell adhesion and reduces cell motility. The right panel represents the disorganised fibrils of the woozy mouse tendon both histologically and ultrastructurally. It also represents the damage to the tenocytes

The inferences we made by bioinformatic analysis about a perturbation of the ECM were confirmed by functional studies. In fact, using DQ-collagen, we could show that ECM remodelling and matrix internalization are impaired in patient cells. We also provided evidences that cell-cell and cell-ECM interactions are altered, thus affecting cell attachment and motility. Finally, our data were corroborated by the TEM analysis of the ECM produced in woozy mice at the MTJ, where tendons connect to muscles. Qualitative analysis showed an organisation of the collagen to form long fibrils with a regular striation in the control animals, whereas in the woozy mouse the arrangement of collagen fibrils in well-aligned fibre bundles was impaired and the ECM showed areas of degenerative disruption. The woozy mouse carries a mis-splicing between exon 7 and ETn retrotransposon leading to the formation of a chimeric transcript of the first 7 exons linked to the transposon. This transcript incorporates a stop codon in frame after 96 bases within the transposon generating a truncated protein that is probably unstable and degraded [38]. Therefore, although the mutations in the fibroblasts of the patient and woozy mice are different, both lead to loss of Sil1. Finally, to best summarise the key experiments and main results of this study, a cartoon is shown in Fig. 7.

ECM is a dynamic structure constituting a large volume of our brain [13, 39]. It generally consists of scaffolding elements such as collagens and amorphous elements such as proteoglycans. Multiple enzymes from the class of metalloproteases and disintegrins, as well as tissue inhibitors of metalloproteinases (TIMPs), are involved in the continuous maturation and remodelling of the matrix with the goal of adapting it to tissue needs [13, 40]. ECM plays important roles in the proliferation, differentiation, and migration of neurons and the establishment of proper connections during brain development [14]. In the adult brain, ECM modulates neuronal activities by acting on dendritic spines and participating in the distribution of neurotransmitters, neurotrophic factors, and nutrients. These actions are also possible because the ECM of the brain is particularly rich in proteoglycans [39]. ECM alterations have been associated to numerous neurodegenerative diseases including Alzheimer’s disease, Huntington’s disease (HD), Parkinson’s disease (PD), epilepsy and ataxia [13, 41]. Spinocerebellar ataxia type 1 (SCA1) is caused by polyglutamine expansion in the Ataxin 1 (ATXN1) gene [42]. A gene expression study in a mouse model of ATXN1 reported changes in the expression of many ECM genes and functionally related genes, including collagens, laminin, integrins, matrix metalloproteinases (MMPs), and disintegrins, suggesting that ECM might be a relevant pathogenetic element in cerebellar ataxias [13, 43]. The expression of ECM genes involved in synapses organization (e.g. Cbln1) was also affected. This gene expression pattern is reminiscent of the one we identified in MSS; it is worth noting that we also revealed alterations in the expression of 5 different semaphorin genes (SEMA3A, SEMA3B, SEMA3C, SEMA5A, SEMA6A). Among the transcription factors involved in the regulation of ECM is the aryl hydrocarbon receptor (AhR)/AhR nuclear translocator (ARNT). In a mouse model of liver fibrosis, AhR activation was shown to trigger the expression of several MMPs and collagens [44]. In line with this, we revealed a downregulation of ARNT2, a possible upstream event contributing to the downregulation of many ECM genes. Note that ARNT2 is neuroprotective in brain injury and is necessary in postnatal brain growth [45, 46].

Analogously to brain, ECM is important in myogenesis process and more in general in muscle maintenance [15, 17]. Muscle consists of post-mitotic cells, and its regeneration relies on satellite cells located in niches containing a specific composition of ECM [17]. This matrix participates in satellite cell self-renewal, proliferation and differentiation, therefore any change in ECM composition can affect muscle fibre regeneration. For example, the change in ECM composition during aging has been associated with the development of sarcopenia [47]. The importance of ECM in skeletal muscle is also underlined by disease generated by mutations in ECM genes especially collagens and laminin or in those affecting ECM receptors [17, 48, 49]. Mutations of collagen VI (COL6) is the cause of different forms of myopathy called Ullrich congenital muscular dystrophy, Bethlem myopathy, limb-girdle muscular dystrophy and myosclerosis [15, 50]. Interestingly, we identified six collagen genes with altered expression, including COL6A1.

ECM disturbance has also been implicated in the development of cataracts, another hallmark clinical manifestation of MSS [51]. Specifically, versican mutations underlie Wagner syndrome, a rare disease characterized by several ocular changes, including vitreopathy, myopia and cataracts [52]. Versican is overexpressed in MSS and might contribute to lens fibrosis and thus cataract. Besides ECM, we would like to underscore that the top downregulated gene in our transcriptomic is ALDH1A1 (Aldehyde dehydrogenase 1 family member a1 also known as Retinal dehydrogenase 1). The knock out of ALDH1A1 is sufficient to cause cataract in a transgenic mouse [53].

Several skeletal abnormalities, such as spinal deformities and short stature, have been described in patients with MSS [2]. Cartilage ECM is an essential component of endochondral ossification [16], therefore conditions affecting cartilage can have important consequences on bone formation. More than one hundred diseases, known as chondrodysplasias, depend on alterations in cartilage components [54]. Cartilage aberrations depend mainly on ER stress and collagen gene mutations. Patients with chondrodysplasias, similar to MSS, have several bone deformities, including short stature. Interestingly, both biglycan and aggrecan, two proteoglycans altered in MSS, have been associated with spondyloepimetaphyseal dysplasia, spondyloepiphyseal dysplasia, and chondrodysplastic dwarfism [55, 56].

Finally, we propose a hypothesis on the mechanism responsible for ECM changes in MSS. To this end, we emphasize that several studies have reported a bidirectional relationship between ER stress and ECM deposition/composition. For example, the transmembrane hyaluronidase TMEM2 by digesting high molecular weight hyaluronan in the ECM protect the cells from ER stress [57]. A pathological role of ER stress and the presence of UPR-promoted ECM accumulation have been reported in open-angle glaucoma [58]. PERK, the main UPR branch in MSS, is required for cell adaptation to the stiffness levels of the ECM [59]. While ATF4, the key UPR-induced transcription factor, plays an important role in the production of matrix to maintain bone homeostasis [60]. These evidences suggest that the UPR is an integrated signalling and transcription circuitry related to the production/quality and functions of ECM.

## Conclusions

In conclusion, our transcriptomic analysis identified ECM as the main injured cellular compartment in MSS. The ECM plays a key role in the development, differentiation, and maintenance of different cell types. There is ample evidence that alterations in ECM affect the proper functioning of the central nervous system, skeletal muscle, bone, and lens. On this basis, we propose that aberrant ECM may be a key pathological feature of MSS and may help explain most clinical manifestations.

### Electronic supplementary material

Below is the link to the electronic supplementary material.

Supplementary Material 1

Supplementary Material 2

Supplementary Material 3

Supplementary Material 4

Supplementary Material 5

Supplementary Material 6

Supplementary Material 7

## Data Availability

The data and materials in this article are available with the agreement of corresponding authors.

## Abbreviations

ATF4: Activating transcription factor 4
ATF6: Activating transcription factor 6
CHOP: C/EBP homologous protein
ALDH1A1: Aldehyde dehydrogenase 1 family member a1
ARNT: Aryl hydrocarbon receptor (AhR)/AhR nuclear translocator
COL6: Ataxin 1 (ATXN1); collagen VI
CTCF: Corrected total cell fluorescence
DE: Differentially expressed
DQ: Dye-quenched
ER: Endoplasmic reticulum
ERAD: ER-associated degradation
eIF2α: Eukaryotic translation initiation factor 2 A
ECM: Extracellular matrix
FC: Fold change
FDR: False discovery rate
FFT: Fast fourier transform
GO: Gene ontology
GAPDH: Glyceraldehyde-3-phosphate dehydrogenase
HD: Huntington’s disease
BiP: Immunoglobulin binding protein
IGF-1: Insulin like growth factor 1
IPA: Ingenuity Pathways Analysis
IRE1: Inositol-requiring enzyme 1
MSS: Marinesco-Sjögren syndrome
mTOR: Mammalian target of rapamycin
MMPs: Matrix metalloproteinases
MTJ: Myotendinous junction
PD: Parkinson’s disease
PI3K: Phosphatidylinositol 3-kinases
NDHF: Primary human dermal fibroblast
PERK: PRK-like endoplasmic reticulum kinase
qPCR: Quantitative real-time PCR
SCA1: Spinocerebellar ataxia type 1
SEMA3A, SEMA3B,SEMA3C, SEMA5A, SEMA6A: semaphorin genes
TEM: Transmission electron microscopy
TIMPs: Tissue inhibitors of metalloproteinases
UPR: Unfolded protein response
