# Supplementary figures and images for: Sil1-deficient fibroblasts generate an aberrant extracellular matrix leading to tendon disorganisation in Marinesco-Sjögren syndrome
Source: J Transl Med. 2024 Aug 23;22:787. doi: 10.1186/s12967-024-05582-0 (PMC11342654; doi:10.1186/s12967-024-05582-0)

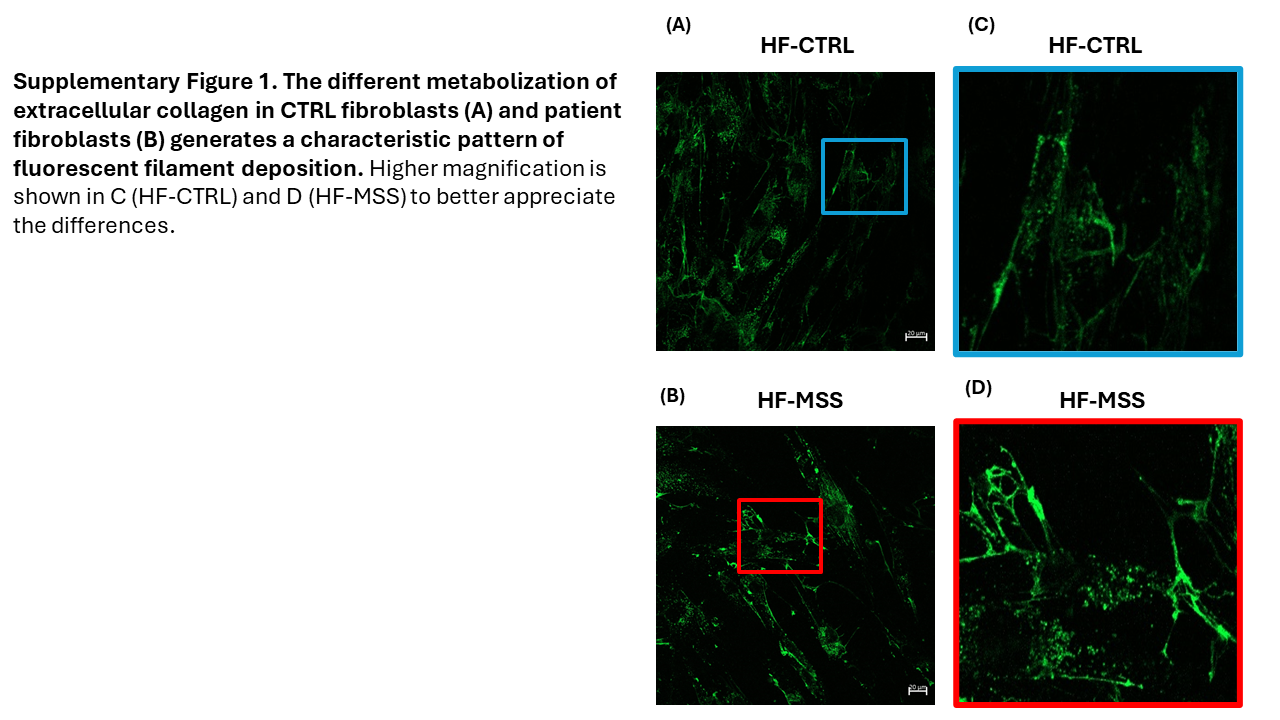

Supplement: Supplementary file 7 — Supplementary Material 7 [file 12967_2024_5582_MOESM7_ESM.tif]
